# Comparative Analysis of Clinical Features of Type 2 Diabetes Mellitus Between Men and Women

**DOI:** 10.7759/cureus.84472

**Published:** 2025-05-20

**Authors:** Tanzeela Nawaz, Veneeza Nawaz, Tasmiya Khurram, Shehzadi Malaika Munsif, Saleha Waheed, Subhana Moin, Adnan Anwar, Atif A Hashmi

**Affiliations:** 1 Internal Medicine, Liaquat College of Medicine and Dentistry, Karachi, PAK; 2 Dentistry, Hamdard College of Medicine and Dentistry, Karachi, PAK; 3 Physiology, Hamdard College of Medicine and Dentistry, Karachi, PAK; 4 Internal Medicine, Sindh Government Hospital, Karachi, PAK; 5 Pathology, Liaquat National Hospital and Medical College, Karachi, PAK

**Keywords:** diabetes mellitus type 2, diabetes symptoms, non-smokers, random blood sugar (rbs), smokers

## Abstract

Objective

Type 2 diabetes mellitus (T2DM) is a chronic metabolic disorder characterized by insulin resistance and hyperglycemia. While its prevalence is rising globally, differences in clinical presentation between genders remain underexplored. This study aims to compare the demographic, physiological, and symptomatic profiles of T2DM between male and female patients.

Methodology

This cross-sectional study was conducted in a secondary care hospital using a non-probability convenience sampling method. The duration of the study was about six months, from April 1, 2024, to September 30, 2024. This study included 400 patients diagnosed with T2DM, comprising 244 males and 156 females. Data were collected through structured questionnaires and clinical assessments, including demographic details, physiological parameters, comorbidities, and renal, ocular, respiratory, psychological, and gastrointestinal symptoms. Statistical analysis was performed using the chi-square test and Mann-Whitney test, with a p-value <0.05 considered significant.

Results

The study findings showed that males exhibited significantly higher weight (p<0.001) and heart rate (p<0.001), while females had significantly higher random blood sugar levels (p<0.001). Females were more likely to belong to the middle socioeconomic class, whereas males had a higher history of smoking (p<0.001). Significant gender-based differences were observed in symptoms such as frequent urination (p<0.001), blurry vision (p<0.001), bilateral edema (p=0.007), dyspnea (p=0.012), burning foot pain (p<0.001), muscular cramps (p<0.001), fatigue (p=0.005), mood changes (p=0.001), and increased thirst (p<0.001).

Conclusion

This study concluded that significant gender-based differences were observed in various clinical characteristics and symptoms among patients with T2DM. Male patients were found to have a higher mean weight and heart rate, while female patients exhibited higher levels of random blood sugar, as well as a higher prevalence of certain symptoms such as blurry vision, bilateral edema, difficulty breathing, and chest tightness.

## Introduction

Globally, diabetes mellitus (DM) impacts approximately 422 million people, directly causing 1.5 million deaths annually and indirectly contributing to 17.5 million more [1]. Type 2 DM (T2DM), which comprises 90-95% of all diabetes cases, arises from insulin resistance and a gradual decline in β-cell function and mass. Since the risk of T2DM is closely related to environmental, dietary, and lifestyle factors, addressing these risk factors through early lifestyle changes is the most effective approach to reducing its prevalence and associated mortality [1].

The global prevalence of obesity and T2DM is steadily increasing, driven by the growing adoption of lifestyles characterized by low physical activity and high-calorie diets, especially in lower-income and developing nations. Projections estimate that T2DM cases will rise from 415 million to 642 million by 2040 [2].

Smoking is a major risk factor for cardiovascular disease (CVD), significantly contributing to the overall cardiovascular burden [3]. In individuals with T2DM, smoking further elevates the risk of macrovascular complications [3]. The 2014 Surgeon General’s Report states that active smokers have a 30-40% higher risk of developing T2DM compared to non-smokers, highlighting the importance of smoking cessation as a critical public health measure to address the global diabetes epidemic [4]. The World Health Organization identifies smoking as a preventable risk factor for T2DM and advocates for smoking avoidance or cessation as part of their lifestyle recommendations [5]. However, the American Diabetes Association does not currently recognize smoking as a modifiable risk factor for diabetes or recommend considering smoking status as a criterion for diabetes screening [6].

Numerous epidemiological studies have identified links between cigarette smoking and the onset of T2DM [7]. In one study involving postmenopausal women in the US, those who smoked an average of 16.2 cigarettes per day had a 1.28-fold higher risk of developing diabetes (95% CI: 1.20, 1.36). However, this risk decreased with smoking cessation; after 10 years of quitting, their risk of diabetes was comparable to that of individuals who had never smoked [8].

The severity of symptoms varies based on the type and duration of diabetes. Some individuals, especially those in the early stages of type 2 diabetes, may not show any symptoms. In contrast, patients, particularly children with severe hyperglycemia and complete insulin deficiency, may exhibit symptoms such as frequent urination, excessive thirst, increased appetite, weight loss, and blurred vision. If not properly managed, diabetes can lead to complications such as diabetic ketoacidosis or, less commonly, a hyperosmolar non-ketotic state, which can result in confusion, coma, and potentially death if left untreated [9,10].

Although men generally have a higher overall prevalence of diabetes, type 2 diabetes is more common among women [11]. Gender differences in diabetes incidence also vary across different stages of reproductive life: more males tend to develop diabetes before puberty, while more females are affected after menopause and in older age. Despite the higher rate of type 2 diabetes in women, men are more prone to forms of diabetes associated with diabetic ketoacidosis or ketosis [12]. Women typically have a natural protection against ketoacidosis, which can become ineffective in conditions of low estrogen or prolonged ovulation [12].

T2DM presents with a wide range of clinical features that may vary by gender due to physiological, hormonal, and behavioral differences. Understanding these gender-specific patterns can aid in early detection, personalized care, and better management strategies. The objective of this study was to compare the clinical characteristics, comorbidities, and symptom profiles of T2DM between male and female patients to identify significant gender-based differences.

## Materials and methods

This cross-sectional study was conducted in a secondary care hospital using a non-probability convenience sampling method. The ethical approval was obtained from the Ethical Review Board of the concerned hospital. The duration of the study was about six months, from April 1, 2024, to September 30, 2024. By using open size Epi software (Centers for Disease Control and Prevention, Atlanta, GA) for sample size calculation, the prevalence of T2DM was 40.0%, as in a previously published study margin of error is 5%, and an interval of 95% [13]. The calculated sample size was 369 patients. This study included 400 type 2 diabetic patients aged 40-65 years of both genders, comprising 244 male diabetic patients and 156 female diabetic patients. Participants must have stable glycemic control, as indicated by recent medical records, and should not have had any acute illness or hospitalization in the past three months. The study excluded individuals with type 1 diabetes, hypoglycemia, prior surgical procedures, or those undergoing chemotherapy; those with a history of substance abuse other than smoking; and patients with severe systemic diseases such as advanced cardiovascular disease, chronic kidney disease, or active malignancy.

Patients were recruited from outpatient clinics after providing written informed consent. Clinical evaluations assessed glycemic control and T2DM-associated complications, using glycosylated hemoglobin (HbA1c) and random blood sugar (RBS) as primary markers. Complication assessments included cardiovascular evaluations via physical exams (blood pressure, heart rate) and laboratory analyses (lipid profiles). Demographic data, such as age, sex, height, weight, BMI, dietary habits, and smoking status, were collected using structured questionnaires. Socioeconomic status (SES) of participants was categorized into three groups based on self-reported information regarding household income, education level, and occupation: low, middle, and high. The presence of diabetes-related complications, including neuropathy, nephropathy, and retinopathy, was examined along with any history of cardiovascular disease. Medical history and sleep disturbances, including insomnia and unusual sleep behaviors, were also recorded. Symptoms of dry eyes were identified based on ocular discomfort, such as irritation, inflammation, blurred vision improving with blinking, and increased tear production. Additionally, heart rate and RBS levels were measured to provide further clinical context.

Data were analyzed using Statistical Product and Service Solutions (SPSS, version 20; IBM SPSS Statistics for Windows, Armonk, NY). The socio-demographic details, such as gender, comorbidities, and clinical symptoms related to T2DM, were reported in frequencies and percentages. Quantitative variables such as age, weight, height, BMI, heart rate, and RBS were represented as means with standard deviations. A chi-square test was applied to examine the association of ocular, respiratory, urinary, psychological, and gastrointestinal symptoms in T2DM between males and females. Additionally, the Mann-Whitney test was used to analyze the relationship between the means of demographic variables in T2DM between males and females. A p-value < 0.05 was considered statistically significant.

## Results

This study included 400 patients with type 2 diabetes, comparing male and female patients. The mean age of male patients was 57.6 ± 15.7 years, while that of female patients was 55.9 ± 14.9 years, showing no statistically significant difference (p=0.146). Males had a significantly higher mean weight (72.2 ± 13.9 kg) compared to females (63.05 ± 14.5 kg, p<0.001). Although the average height was greater in males (67.8 ± 11.1 inches) than in females (65.7 ± 6.77 inches), this difference was statistically insignificant (p=0.187). Similarly, body mass index (BMI) was higher in males (26.8 ± 12.9 kg/m²) than in females (23.6 ± 7.78 kg/m²), with a statistically insignificant difference (p=0.088). However, heart rate was significantly elevated in males (88.2 ± 10.4 beats/min) compared to females (81.9 ± 12.0 beats/min, p<0.001). Interestingly, random blood sugar (RBS) levels were significantly higher in females (317.4 ± 115.6 mg/dL) than in males (307.6 ± 104.7 mg/dL), with a statistically significant difference (p<0.001), as presented in Table 1.

**Table 1:** Demographic details of type 2 diabetes patients (n=400) Data have been presented as mean and SD. P-value of <0.05 considered significant.

Variables	Male (n=244) Mean±SD	Female (n=156) Mean±SD	P-value
Age (years)	57.6±15.7	55.9±14.9	0.146
Weight (kg)	72.2±13.9	63.05±14.5	<0.001
Height (inch)	67.8±11.1	65.7±6.77	0.187
BMI (kg/m^2^)	26.8±12.9	23.6±7.78	0.088
Heart rate (beats/min)	88.2±10.4	81.9±12.0	<0.001
Random blood sugar (RBS) (mg/dL)	307.6±104.7	317.4±115.6	<0.001

The association of gender, comorbidities, and socioeconomic status among T2DM patients revealed significant differences between males and females. Age distribution differed notably between genders (p=0.001), with a higher proportion of females (87, 55.8%), in the 30-50-year age group compared to males (90, 36.9%). In contrast, more males were in the older age groups of 51-70 years (9, 38.1%) and 71-95 years (61, 25.0%), compared to females (46, 29.5%, and 23, 14.7%, respectively). Socioeconomic status also showed a significant gender-based difference (p=0.007). There were no significant gender differences in the history of hypertension (p=0.413) or dyslipidemia (p=0.357). However, smoking history showed a highly significant difference between genders (p<0.001), with 186 (76.2%) of males reporting a history of smoking compared to only 14 (9.0%) of females, as presented in Table 2.

**Table 2:** The association of gender, comorbidities, and socioeconomic status in T2DM patients between genders Data have been presented as n (%). A p-value of <0.05 is considered significant.

Variables		Male, n(%)	Female, n(%)	P-value
Age group (years)	30-50	90(36.9%)	87(55.8%)	0.001
	51-70	93(38.1%)	46(29.5%)	
	71-95	61(25.0%)	23(14.7%)	
Socioeconomic status	Low	39(16.0%)	26(16.7%)	0.007
	Middle	131(53.7%)	104(66.7%)	
	High	74(30.3%)	26(16.7%)	
History of hypertension	Yes	169(69.3%)	114(73.1%)	0.413
	No	75(30.7%)	42(26.9%)	
History of dyslipidemia	Yes	176(72.1%)	119(76.3%)	0.357
	No	68(27.9%)	37(23.7%)	
History of smoking	Yes	186(76.2%)	14(9.0%)	<0.001
	No	58(23.8%)	142(91.0%)	

A significant association was observed between gender and several renal, ocular, and respiratory symptoms in T2DM patients. Frequent urination was reported more commonly among males (110, 45.1%) than females (42, 26.9%), showing a statistically significant difference (p<0.001). Blurry vision was significantly more prevalent in females (84, 53.8%) than in males (68, 27.9%) (p<0.001). Bilateral edema was also significantly more frequent in females (84, 53.8%) than males (98, 40.2%) (p=0.007). Furthermore, when examining the severity of bilateral edema, moderate edema was more frequent among females (109, 69.9%) than males (98, 40.2%), while mild edema was more common in males (122, 50.0%) than in females (27, 17.3%) (p<0.001). Dyspnea grading showed a significant difference (p=0.012), with a greater percentage in females (88, 56.4%) experiencing dyspnea while climbing stairs, compared to males (121, 49.6%). Difficulty in breathing was also significantly associated with gender (p<0.001). Additionally, chest tightness or pressure was significantly more common in females (125, 80.1%) than in males (161, 66.0%) (p=0.002). In contrast, no significant gender differences were found in nighttime urination frequency (p=0.720), poor night vision (p=0.094), confusion or difficulty concentrating (p=0.294), and shortness of breath (p=0.245), as presented in Table 3.

**Table 3:** The association of renal, ocular, and respiratory symptoms in T2DM patients between genders Data have been presented as n (%). A p-value of <0.05 is considered significant.

Variables		Male, n(%)	Female, n(%)	P-value
Frequent urination	Yes	110(45.1%)	42(26.9%)	<0.001
	No	134(54.9%)	114(73.1%)	
Urination at night	Three times at night	144(59.0%)	98(62.8%)	0.720
	Two times at night	90(36.9%)	53(34.0%)	
	Every night	10(4.1%)	5(3.2%)	
Blurry vision	Yes	68(27.9%)	84(53.8%)	<0.001
	No	176(72.1%)	72(46.2%)	
Poor night vision	Yes	77(31.6%)	62(39.7%)	0.094
	No	167(68.4%)	94(60.3%)	
Edema, if yes, then	Bilateral	98(40.2%)	84(53.8%)	0.007
	Unilateral	146(59.8%)	72(46.2%)	
If bilateral then	1+ Mild (Both ankles/feet)	122(50.0%)	27(17.3%)	<0.001
	2+ Moderate (Both feet, hands, lower arms, and lower legs)	98(40.2%)	109(69.9%)	
	3+ Severe (Generalized bilateral pitting edema, including both legs, arms, feet and face)	24(9.8%)	20(12.8%)	
Confusion or difficulty concentrating	Yes	95(38.9%)	69(44.2%)	0.294
	No	149(61.1%)	87(55.8%)	
Shortness of breath	Yes	161(66.0%)	94(60.3%)	0.245
	No	83(34.0%)	62(39.7%)	
Dyspnea grading	While climbing stairs	121(49.6%)	88(56.4%)	0.012
	While walking for more than 6 hours a day	79(32.4%)	58(37.2%)	
	While walking for less than 6 hours a day	23(9.4%)	5(3.2%)	
	While at rest	21(8.6%)	5(3.2%)	
Difficulty breathing, if yes	Mild	72(29.5%)	84(53.8%)	<0.001
	Moderate	123(50.4%)	57(36.5%)	
	Severe	49(20.1%)	15(9.6%)	
Chest tightness or pressure	Yes	161(66.0%)	125(80.1%)	0.002
	No	83(34.0%)	31(19.9%)	

The association between psychological and gastrointestinal symptoms among type 2 diabetes patients with both genders revealed that burning pain in the legs or feet was significantly more prevalent in females (115, 73.7%) than in males (125, 51.2%) (p<0.001). Similarly, sensitivity of the feet to touch was reported more frequently in females (64, 41.0%) than in males (57, 23.4%) (p<0.001). Muscular pain or cramps in the legs or feet were universally reported by all females (156, 100.0%), significantly higher than males (206, 84.4%) (p<0.001). Increased thirst was significantly more common in females (116, 74.4%) than in males (73, 29.9%) (p<0.001), while fatigue also showed a significant association with gender, reported by 145 (92.9%) of females compared to males by 203 (83.2%) (p=0.005). Feeling tired and weak was noted in 135 (86.5%) of females, significantly more than 169 (69.3%) of males (p<0.001). Additionally, mood changes were more frequently reported in females (135, 86.5%) than in males (177, 72.5%), showing statistical significance (p=0.001). In contrast, no significant gender differences were found in tingling or numbness in the hands or feet (p=0.079), loss of appetite (p=0.710), and insomnia (p=0.522), as presented in Table 4.

**Table 4:** The association between psychological and gastrointestinal symptoms in T2DM patients between both genders Data have been presented as n (%). A p-value of <0.05 is considered significant.

Variables		Male, n(%)	Female, n(%)	P-value
Tingling or numbness in the hands or feet	Yes	162(66.4%)	90(57.7%)	0.079
	No	82(33.6%)	66(42.3%)	
Burning pain in your legs or feet	Yes	125(51.2%)	115(73.7%)	<0.001
	No	119(48.8%)	41(26.3%)	
Too sensitive feet on touch	Yes	57(23.4%)	64(41.0%)	<0.001
	No	187(76.6%)	92(59.0%)	
Muscular pain or cramps in your legs or feet	Yes	206(84.4%)	156(100.0%)	<0.001
	No	38(15.6%)	0(0.0%)	
Loss of appetite	Yes	150(61.5%)	93(59.6%)	0.710
	No	94(38.5%)	63(40.4%)	
Insomnia	Yes	114(46.7%)	78(50.0%)	0.522
	No	130(53.3%)	78(50.0%)	
Increased thirst	Yes	73(29.9%)	116(74.4%)	<0.001
	No	171(70.1%)	40(25.6%)	
Fatigue	Yes	203(83.2%)	145(92.9%)	0.005
	No	41(16.8%)	11(7.1%)	
Feel tired and weak	Yes	169(69.3%)	135(86.5%)	<0.001
	No	75(30.7%)	21(13.5%)	
Mood changes	Yes	177(72.5%)	135(86.5%)	0.001
	No	67(27.5%)	21(13.5%)	

## Discussion

DM is an endocrine condition resulting from either inadequate insulin production or dysfunctional insulin signalling, which impairs glucose regulation and disrupts the metabolism of carbohydrates, proteins, and fats [14]. Therefore, the present study demonstrated the comparison of clinical features of T2DM patients between both genders.

One of the cross-sectional studies analyzed neurological symptoms in 525 diabetic patients with type 1 and type 2 diabetes, focusing on gender differences. Among the participants, 40% were female, and 60% were male, with mean ages of 50.52 ± 14.8 years for females and 57.36 ± 14.99 years for males, showing a significant age difference (p<0.001). Irritability or mood swings was the most reported symptom, affecting 68.6% of males and 77.6% of females (p=0.022). Significant gender associations were also noted for swelling, confusion, burning pain, and muscle cramps. Additionally, hypertension, dyslipidemia, and smoking influenced some symptoms [15]. These findings were inconsistent with the present study, which indicated the mean age was insignificantly higher in males (57.6 ± 15.7 years) compared to females (55.9 ± 14.9 years, p=0.146). Concerning clinical features, mood changes were more prevalent in females (135, 86.5%) than in males (177, 72.5%), showing a statistically significant difference (p=0.001). Urinary symptoms, muscular pain, tiredness and weakness, increased thirst, and blurred vision were significantly more common in females than the males.

Gender-based discrimination exacerbates environmental mental distress and stress responses, with women being particularly affected. Research suggests that women are more susceptible to the harmful cardio-metabolic consequences of psychological stress, job-related stress, and sleep disorders. This heightened vulnerability may be partly due to unhealthy coping behaviors [16,17]. A sex-specific meta-analysis of epidemiological data found that women of all ages are 40% more likely to suffer from insomnia [18]. Moreover, obesity and, more notably, insulin resistance-related conditions such as impaired glucose metabolism have been associated with disrupted sleep, shorter sleep duration, and poor sleep quality [19]. Another meta-analysis revealed that inadequate sleep (less than five hours) and difficulty maintaining sleep are both linked to an elevated risk of developing diabetes. However, when data were analyzed by gender, the strength of these associations appeared similar in both men and women [19]. In contrast, the present study's findings do not show a statistically significant gender difference in the prevalence of insomnia between groups (114 (46.7%) vs. 78 (50.0%); p=0.522). This contrasts with the cited literature, which reported significantly higher insomnia prevalence in women.

Smoking plays a significant role in health differences between men and women. Over the past decade, smoking rates have increased notably among young women, potentially leading to a higher incidence of smoking-related diabetes in this group [20]. A meta-analysis indicated that the risk of myocardial infarction, a common and serious complication in individuals with diabetes linked to smoking, is approximately 25% higher in women than in men [21]. Similarly, another meta-analysis of cohort studies found that both active and passive smoking are associated with a heightened risk of developing type 2 diabetes in both genders, with no significant difference observed between men and women [21]. In contrast, the present study reveals a strong gender disparity in smoking history among type 2 diabetes patients, with males showing much higher rates of smoking.

A study conducted in Nigeria identified gender-specific differences in cardio-metabolic risk factors, as well as microvascular and macrovascular complications among patients with type 2 diabetes. The study included 210 female participants (52.5%) and 190 male participants (47.5%), with an average age of 60.6 ± 9.93 years. Women were found to have higher rates of obesity and hypertension. Interestingly, a greater proportion of men with type 2 diabetes achieved LDL cholesterol treatment goals compared to women (69.5% vs. 59.0%, p<0.05) [22]. As far as the present study is concerned, the mean age was slightly lower: 57.6 ± 15.7 years for males and 55.9 ± 14.9 years for females, with no statistically significant gender difference (p=0.146). The present study found that males had a significantly higher mean weight (72.2 ± 13.9 kg) compared to females (63.05 ± 14.5 kg; p<0.001). Although males also had a higher BMI (26.8 ± 12.9 kg/m² vs. 23.6 ± 7.78 kg/m²), this difference was not statistically significant (p=0.088). However, the present study found no statistically significant gender difference in the history of hypertension (p=0.413).

This study had a few limitations. This study is limited by its cross-sectional design, which prevents causal inferences, and the reliance on self-reported symptoms, which may introduce reporting bias. Additionally, the study was conducted in a single geographic area, which may affect the generalizability of the findings. Future research should include longitudinal studies to assess temporal relationships and explore underlying mechanisms. Broader, multicenter studies with diverse populations are recommended to enhance the generalizability of results. Routine screening for systemic and psychological symptoms in T2DM patients should be integrated into clinical practice.

## Conclusions

This study concluded that significant gender-based differences were observed in various clinical characteristics and symptoms among patients with T2DM. Male patients were found to have a higher mean weight and heart rate, while female patients exhibited higher levels of RBS, as well as a higher prevalence of certain symptoms, such as blurry vision, bilateral edema, difficulty breathing, and chest tightness. Psychological and gastrointestinal symptoms, including increased thirst, fatigue, and mood changes, were also more common in females.
